# Longitudinal wall motion of the common carotid artery can be assessed by velocity vector imaging

**DOI:** 10.1111/j.1475-097X.2010.00976.x

**Published:** 2011-01

**Authors:** Sara Svedlund, Li-Ming Gan

**Affiliations:** 1 The Department of Clinical Physiology, Sahlgrenska University HospitalGothenburg; 2 Department of Clinical and Molecular Medicine, Institution of Medicine, Sahlgrenska Academy at the University of GothenburgGothenburg; 3 BioScience, AstraZeneca R&DMölndal, Sweden

**Keywords:** arterial stiffness, cardiovascular disease, carotid artery ultrasound, longitudinal displacement, surrogate marker

## Abstract

Velocity vector imaging (VVI) is novel ultrasound image analysis software, enabling simultaneous evaluation of longitudinal and radial tissue motion. This study aimed to investigate the possible usefulness of VVI in evaluating the longitudinal vessel wall movement of the common carotid artery (CCA). Sixteen healthy volunteers and 16 patients with established coronary artery disease (CAD) were included in the study. CCA was scanned and standard B-mode ultrasound images were analysed off-line with VVI. In healthy volunteers, total longitudinal displacements (tLoD) of the right and left CCA were similar, as were the movements of the near- and far wall of the right CCA. The CAD group showed significantly lower tLoD compared to the healthy volunteers (0·543 ± 0·394 versus 0·112 ± 0·074, *P*<0·0001). VVI is a highly feasible technique in assessing longitudinal CCA wall motion, which may be of potential relevance as a novel vascular biomarker.

## Introduction

Arterial stiffness measured with different techniques is well known to be of importance for future cardiovascular morbidity and mortality (Franklin *et al.*, 1999; Weber *et al.*, 2004; Dolan *et al.*, 2006; Willum-Hansen *et al.*, 2006). The golden standard method to assess arterial stiffness is pulse wave velocity (PWV) (Laurent *et al.*, 2006), which measures the time of a pulse wave along an arterial segment and relies on transfer functions for pressure estimation. PWV has been shown to be of prognostic relevance in different patient cohorts (Guerin *et al.*, 2001; Boutouyrie *et al.*, 2002; Cruickshank *et al.*, 2002; Mattace-Raso *et al.*, 2006). Local methods focusing on the diameter change during heart cycles, i.e. the radial movement has also been extensively studied in terms of local strain, distensibility and compliance. However, the longitudinal arterial movement has gained little research focus, and its clinical implications remains to be elucidated.

Increased intima-media thickness is a well-established marker of atherosclerotic diseases and provides a predictive value for cardiovascular events (O’Leary *et al.*, 1999; Ali *et al.*, 2006). In addition to the structural information, the carotid images are usually recorded in real time, which make it possible to study common carotid artery (CCA) wall motion, typically in the radial direction using e.g. M-mode. The concept to combine simultaneous morphological and functional measurement is highly attractive, especially when the imaging procedure is highly feasible, non-invasive and inexpensive.

Recently, (Persson *et al.*, 2003) developed a new ultrasound-based technique with the ability to assess radial, as well as longitudinal CCA wall motion in man. With ultrasound technique, they demonstrated a distinct longitudinal vessel wall movement in healthy subjects (Cinthio *et al.*, 2006). However, little is known about the longitudinal movements’ clinical correlates and possible usefulness. Velocity vector imaging (VVI) is new ultrasound software, based on multiple M-mode evaluations and speckle-tracking technique. The software has been used to study ventricle dyssynchronies of the heart (Cannesson *et al.*, 2006). VVI allows simultaneous evaluation of longitudinal and radial velocity, strain, strain rate and displacement. In this study, we aimed to investigate the applicability and reproducibility of VVI in assessment of longitudinal CCA wall motion. Additionally, the potential difference in longitudinal displacement between healthy volunteers and a group of patients with established coronary artery disease (CAD) was investigated.

## Materials and method

### Study population

Sixteen young healthy volunteers free of symptomatic cardiovascular disease and any medications were recruited for study participation. As a control group, 16 patients with established atherosclerotic CAD were recruited with the inclusion criteria of prior CABG operation. All subjects gave their informed and written consent to study participation. The study was approved by the Local Ethics Committee in Gothenburg.

### Carotid artery ultrasound examinations

Carotid artery B-mode ultrasound was conducted by an experienced sonographer according to a standardized protocol over the distal CCA. All examinations were performed with the study subject in the supine position after 10 min of rest. The Acuson Sequoia 512 ultrasound system (Siemens Medical Solutions Inc., Mountain View, USA), equipped with an 8- MHz transducer was used. CINE-looped images of consecutive cardiac cycles of the distal CCA were stored for later offline analysis. Intima-media thickness (IMT) was defined as the distance from the lumen-intimal interface to the medial-adventitial border and measured approximately one centimetre proximal to the bulb of the CCA far wall (Wendelhag *et al.*, 1991). The presences of plaques were evaluated in long-axis view of the carotid bifurcation by manual delineation. As conventional local stiffness measurements, CCA strain, based on the radial diameter change during a heart cycle, was calculated using the formula: 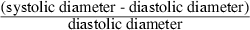
and carotid stiffness index was calculated as: 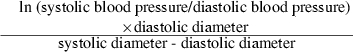
where ln is the natural logarithm and blood pressure was measured in the brachial artery by conventional oscillometric approach as an estimate (Kawasaki *et al.*, 1987).

### Velocity vector imaging of the common carotid artery

**Figure 1:**
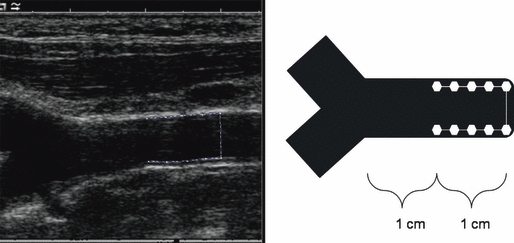
The left picture shows delineation of the lumen vascular borders using the VVI tool in a standard common carotid artery (CCA) image. The schematic picture to the right illustrates the guiding points in the near- and far wall of the CCA proximal to the carotid bulb.

**Figure 2:**
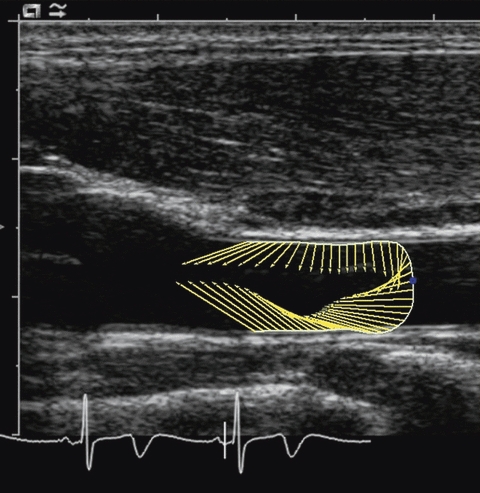
The image shows the common carotid artery (CCA) wall motion at the specific time point shown in the ECG recording. The vectors indicate the direction and magnitude of the CCA wall motion in the longitudinal and radial direction.

All VVI-measurements were made offline at a workstation by two other independent observers. VVI software (Research Arena 2; TomTec imaging systems GmbH, Unterschleissheim, Germany) was used to derive vessel wall velocity, strain, strain rate and displacement of both the near- and far wall of the right and left CCA. Further, the radial movement in terms of velocity and displacement was studied. Delineation of the lumen-vessel wall border was conducted using the leading-to-leading edge principle; edges were outlined approximately 1 cm distal to the carotid bulb in the right and left CCA. Five guiding points were equally distributed (0·25 cm apart) within a 1- cm segment both in the near wall and far walls, i.e. a total of ten measuring points were used. With available software, it is possible to evaluate the vessel wall movements of single wall segments; the outlined segments corresponding to the near- and far wall of the CCA were selected. The virtual transducer applied in the software was placed above the near wall. Figure 1 illustrates an outlined measurement. Software settings were positioned to cover peak systole and peak diastole values during one complete cardiac cycle. To be able to quantify the longitudinal movement, we calculated the total longitudinal displacement (tLoD) during a cardiac cycle as the sum of absolute values of maximal systolic plus the maximal diastolic displacements. Figure 2 displays an example of the processed measurement with the vectors indicating the movement of the CCA walls in the longitudinal direction.

### Reproducibility measurements

We evaluated intra-observer and inter-observer variability of the measurements at separate occasions at least 4 weeks apart. For intra- and inter-observer variability, coefficients of variance were calculated using the formula: 
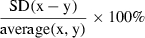
where x and y represent values of the first and the second measurement, respectively.

### Statistics

A *P*-value of <0·05 was considered significant. To determine differences in vessel wall motion between different wall segments and the left and right side, a Student’s paired *T*-test was used. To study differences between genders and healthy and diseased subjects, Student’s *t*-test was performed.

## Results

### Healthy volunteers

Table 1 shows clinical data of the study groups with comparison by gender. The 16 healthy subjects were aged 25·4 ± 5·1 years (range 20–38 years) and were normotensive. They all displayed a distinct measureable longitudinal movement during cardiac cycles. There was no significant difference between far wall mean tLoD of the right CCA (mean 0·543 ± 0·394 mm, range 0·090–1·516 mm) and the left CCA (mean 0·401 ± 0·274 mm, range 0·036 ± 0·824 mm). There was no difference in tLoD between men and women (0·532 ± 0·460 mm versus 0·557 ± 0·325 mm, *P* = NS). Table 2 displays the VVI-generated variables of the CCA with comparisons of both the different walls in the right CCA and comparisons of the right and left CCA. The radial movement amplitude was similar to that of the longitudinal.

**Table 1:** Clinical characteristics of the study groups with gender comparisons

	Healthy volunteers (*n* = 16)			CAD-group (*n* = 16)		
		
	Males	Females	*P*	Males	Females	*P*
Gender (%)	56·3	43·7	NS	60·0	40·0	NS
Age (years)	26·8 ± 6·5	23·6 ± 6·9	NS	66·8 ± 9·6	70·2 ± 3·1	NS
HR (bpm)	54·4 ± 6·1	62·3 ± 8·9	NS	65·1 ± 8·4	57·3 ± 9·9	NS
BMI (kg m^−2^)	24·2 ± 3·2	21·5 ± 1·3	0·05	26·7 ± 1·6	21·6 ± 10·9	NS
SBP (mmHg)	112·2 ± 8·3	101·4 ± 14·4	NS	150·9 ± 10·3	155·8 ± 37·5	NS
DBP (mmHg)	66·9 ± 7·5	63·0 ± 8·4	NS	86·6 ± 11·1	76·6 ± 6·1	NS
PP (mmHg)	46·3 ± 11·6	46·0 ± 9·6	NS	64·4 ± 17·7	79·2 ± 31·7	NS
CCA dS (cm)	0·64 ± 0·06	0·61 ± 0·04	NS	0·65 ± 0·07	0·67 ± 0·11	NS
CCA dD (cm)	0·57 ± 0·06	0·54 ± 0·02	NS	0·61 ± 0·07	0·61 ± 0·11	NS
CCA IMT (cm)	0·029 ± 0·008	0·027 ± 0·004	NS	0·057 ± 0·010	0·060 ± 0·011	NS
CCA strain	0·122 ± 0·052	0·142 ± 0·055	NS	0·067 ± 0·028	0·111 ± 0·068	NS
CCA stiffness index	5·8 ± 3·9	3·7 ± 0·8	NS	9·34 ± 4·39	7·53 ± 2·56	NS

Values are displayed as means ± SD. HR, heart rate; BMI, body mass index; SBP, systolic blood pressure; DBP, diastolic blood pressure; PP, pulse pressure; CCA dS, common carotid artery diameter in systole; CCA dD, common carotid artery diameter in diastole; IMT, intima-media thickness.

**Table 2:** Comparisons between various VVI-derived CCA wall motion measurements in healthy volunteers

	RCCA			LCCA	
				
	NW	FW	*P*-value^a^	FW	*P*-value^b^
Longitudinal variables					
Velocity S (cm s^−1^)	0·278 ± 0·230	0·290 ± 0·230	NS	0·166 ± 0·116	NS
Velocity D (cm s^−1^)	0·168 ± 0·097	0·173 ± 0·093	NS	0·134 ± 0·093	NS
Strain S (%)	7·519 ± 5·412	6·991 ± 6·274	NS	4·732 ± 3·920	NS
Strain D (%)	1·443 ± 1·722	1·696 ± 1·632	NS	1·159 ± 1·708	NS
Strain rate S (l s^−1^)	0·422 ± 0·348	0·419 ± 0·376	NS	0·234 ± 0·162	NS
Strain rate D (1 s^−1^)	0·283 ± 0·192	0·266 ± 0·202	NS	0·205 ± 0·129	NS
Displacement S (mm)	0·457 ± 0·347	0·474 ± 0·356	NS	0·332 ± 0·262	NS
Displacement D (mm)	0·075 ± 0·086	0·069 ± 0·088	NS	0·069 ± 0·180	NS
Total displacement (mm)	0·531 ± 0·372	0·543 ± 0·394	NS	0·401 ± 0·274	NS
Radial variables					
Velocity S (cm s^−1^)	0·100 ± 0·071	0·100 ± 0·071	NS	0·070 ± 0·061	NS
Velocity D (cm s^−1^)	0·133 ± 0·093	0·080 ± 0·064	0·005	0·053 ± 0·050	0·04
Displacement S (mm)	0·429 ± 0·314	0·162 ± 0·160	0·001	0·109 ± 0·120	NS
Displacement D (mm)	0·027 ± 0·041	0·030 ± 0·031	NS	0·037 ± 0·062	NS

a *P*-value indicates significance between near wall and far wall of the RCCA.

b *P*-value shows significance between RCCA and LCCA far walls.

### Patients with established coronary artery disease

**Figure 3:**
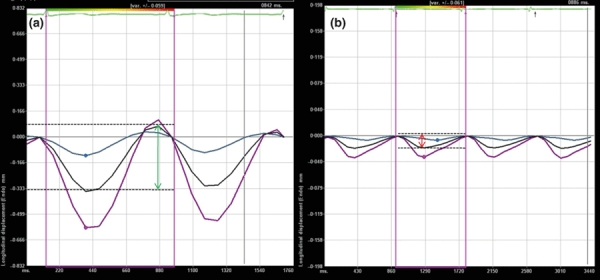
Shows detailed examples from the output of the VVI-software. Displayed are the longitudinal displacement curves during heart cycles. The blue and purple curves correspond to the common carotid artery far wall; the black curve is the average of the blue and purple curves. The purple vertical lines denote the measured cardiac cycle. The ECG recording can be seen above the displacement curves. (a) Displays a normal longitudinal wall motion of the CCA far wall in a healthy volunteer. The green arrow indicates tLoD, which in this patient was found to be 0·438 mm. (b) Shows a patient with low tLoD. The red arrow indicates the tLoD to be 0·019 mm.

Patients who previously underwent a CABG and hence are considered to exhibit established CAD showed significantly lower tLoD of both the near- and far wall of the CCA as shown in Table 3. No significant difference between men and women in tLoD could be found (0·093 ± 0·078 versus 0·126 ± 0·073, *P* = NS). Of the patients who underwent CABG, 50% (8/16) were on ongoing treatment with ACE inhibitors, 81·3% (13/16) on beta blockers, 68·8% (11/16) on statins, 75% (12/16) on aspirin and 37·5% (6/16) were treated with clopidogrel. Further, 12 of the 16 patients exhibited plaques in the CCA bifurcation; the average plaque area in this study group was 0·151 ± 0·209 cm. Figure 3 illustrates detailed examples from the software of tLoD in healthy volunteers and patients with CAD.

**Table 3:** Longitudinal displacements in healthy volunteers and patients with CAD

	Healthy subjects (*n* = 16)	CAD-patients (*n* = 16)	*P*-values
Longitudinal variables			
Displacement S NW (mm)	0·457 ± 0·347	0·078 ± 0·065	<0·0001
Displacement D NW (mm)	0·075 ± 0·086	0·032 ± 0·062	NS
tLoD NW (mm)	0·531 ± 0·372	0·101 ± 0·079	<0·0001
Displacement S FW (mm)	0·474 ± 0·354	0·093 ± 0·075	<0·0001
Displacement D FW (mm)	0·069 ± 0·088	0·019 ± 0·024	0·04
tLoD FW (mm)	0·543 ± 0·394	0·112 ± 0·074	<0·0001

S, systole; D, diastole; NW, near wall; FW, far wall; tLoD, total longitudinal displacement.

### Reproducibility measurements

Intra-observer reproducibility of the total longitudinal displacement was found to be 10·5% for the CCA far wall and 12·2% for the CCA near wall. Table 4 shows the intra-observer and inter-observer variability at repeated measurements.

**Table 4:** Intra- and inter-observer coefficients of variance

	Near wall	Far wall
Intra-observer (%)	12·2	10·5
Inter-observer (%)	18·1	9·1

## Discussion

This study shows tLoD to be measurable with VVI-technique. Further, our data shows the longitudinal movement of the CCA near- and far wall to be of similar magnitude in healthy volunteers. Additionally, no differences in movements could be seen between the right and left CCA. Patients with CAD displayed significantly lower total longitudinal displacement compared to healthy volunteers. The intra-observer and inter-observer reproducibility of the right CCA far wall tLoD was found to be 10·5% and 9·1%, respectively.

### Measuring longitudinal displacement with velocity vector imaging technique

As the arterial system is complex and susceptible to a variety of different physiological conditions, characterization of arterial stiffness has faced practical problems (O’Rourke & Mancia, 1999). The longitudinal wall motion is known to be very small studied in experimental models (Patel *et al.*, 1961; Patel & Fry, 1966) and has not been under any specific research focus. This is probably due to lack of an adequate tool to assess this small movement. Now, VVI-technique provides a potential method, which is readily available. We found a visually good tracking ability of wall movements, which is confirmed by previous experimental validation of the VVI-technique (Pirat *et al.*, 2008). The technique reveals the longitudinal CCA movement of the near- and far wall to be of about the same magnitude in healthy volunteers. Further, also the movements of the right and left CCA are of about the same size. CCA movements in healthy volunteers reveal somewhat large individual variations, and this could be because of both physiological and methodological reasons. Nevertheless, we suggest that the measurements of the small movement must be very carefully conducted in a standardized manner. With the present available VVI-software version and the reproducibility data provided in this study; we calculated the tLoD of the CCA during a heart cycle, which seems to be a robust read-out variable of CCA wall motion. Further, we conducted the measurements on the far wall of the right CCA, usually with best 2D image quality, to provide a standardized measuring concept.

Concerning the VVI-technique, it is based on speckle-tracking technique and multiple M-mode evaluations. In the current VVI setting, the longitudinal wall motion was averaged from a 1-cm segment at the lumen-vessel boundary of the CCA far wall. There is thus no particular region of interest (ROI) that is evaluated, but several speckles that are integrated and then averaged. This averaging approach using VVI is probably not sensitive enough to detect multiphasic motions at a particular ROI in the vessel wall. However, this probably makes the VVI-technique less susceptible for local variations throughout the CCA. This fact is probably the main reason that the technique launched by Cinthio *et al.* is better suited to study the multiphasic longitudinal wall motion.

### Radial wall motion

Using VVI-technique to evaluate the radial wall movements, it provides the radial velocity and displacement. Radial wall movements in terms of diameter changes are more easily performed by established methods. Our data indicate the radial CCA displacement to be of about equal size compared to the longitudinal movement in the CCA near wall and of about twice the size in the far wall. This finding confirms the data from previous observations (Golemati *et al.*, 2003; Cinthio *et al.*, 2006). Further, in consistence with the findings by (Persson *et al.*, 2003), we found that the CCA near wall had greater radial wall movement than the far wall. However, the nature of this phenomenon still remains to be determined. Several local arterial stiffness methods, most based on ultrasound investigations, relay on the radial diameter changes and often take blood pressure into account. Calculation of the carotid stiffness index is a well-known local measure of arterial stiffness (O’Rourke *et al.*, 2002). As can be confirmed in our study; patients with CAD show increased stiffness index (Hirai *et al.*, 1989).

### Aspects on vessel wall mechanical properties

Considering arterial biomechanics, wall shear stress and the circumferential stress acting on the arterial wall traditionally account for vessel wall remodelling during pathological circumstances. Altered blood flow is known to impact on vessel wall radius (Langille *et al.*, 1989; Sho *et al.*, 2004), whereas blood pressure acts on vessel wall thickness (Courtman *et al.*, 1998; Hu *et al.*, 2007) to adapt with the goal of re-achieving arterial vessel wall homeostasis (Davies, 1995). The fundamental axial stress acting on the vessel in the longitudinal direction is less well studied in comparison, and increasing interest is aimed at evaluation of the biaxial forces in experimental settings. However, axial stretch *in vivo* has been shown to be reduced with increasing wall thickness or hypertension (Wolinsky, 1970; Eberth *et al.*, 2009). Elastin has been proposed to play a crucial role in providing an axial prestretch *in vivo* owing to the fact that elastin is stretched during development (Dobrin *et al.*, 1990). Increased collagen deposition that follows hypertension will consequently result in increased wall thickness and a reduced axial retraction. This becomes evident when calculating the C:E ratio (collagen/elastin) where a larger ratio is clearly associated with lower *in vivo* axial stretch (Wolinsky, 1970; Berry & Greenwald, 1976). It has been shown both theoretically and experimentally that following vessel wall thickening, the axial stress is decreased to larger extent than the circumferential stress. This may suggest that, compared to the traditional radial wall strain, the longitudinal wall motion might be an earlier and more sensitive measure of vascular wall remodelling (Humphrey *et al.*, 2009). Not surprisingly, patients who underwent CABG in the present study display significantly greater IMT than the healthy subjects because of their advanced systemic atherosclerotic disease. This could be one of the important mechanism underlying the lower tLoD in patients who underwent CABG compared to the healthy volunteers.

### Potential clinical values of the CCA longitudinal wall motion

At present, great research efforts are made to define risk markers of early atherosclerotic disease with the ambition to predict future cardiovascular (CV) events. The CCA longitudinal wall motion could be a new potential risk marker with additional values on top of the CCA morphological information. Also, physiological and molecular determinants of tLoD need to be investigated further.

This study showed the longitudinal wall movement in patients with CAD to be significantly decreased in all measured segments compared to healthy volunteers. The comparison was performed to demonstrate potential biological significance of this measure in this very initial study. Obviously, no adjustments for age and traditional CV risk factors were made at this point. Nevertheless, tLoD is highly distinct between the two groups. If tLoD could be used as an independent factor for CV risk, stratification still remains to be addressed in future prospective studies in a relevant CV patient cohort. At this point, the found difference in tLoD could be caused by various factors such as age, blood pressure, CV disease burden and different medications.

In advanced atherosclerotic vascular disease, it is conceivable that the radial as well as the longitudinal wall motion is impaired. However, it is likely that the longitudinal wall motion indices are more sensitive to detect early vessel wall pathology as well as to study adaptive vascular change and possible treatment effects, based on the aforementioned theoretical analysis. The more prominent differences in the longitudinal versus the radial wall motion between CAD and healthy subjects in this initial study may support this hypothesis.

### Study limitations

The VVI software used in this study has the ability to generate peak systolic and diastolic values during a cardiac cycle. As a measuring concept, the software currently serves for cardiac wall motion evaluation, but can most likely be even further developed and specifically adapted for vessel wall function evaluation. The longitudinal CCA movement has previously been shown to be multiphasic (Cinthio *et al.*, 2006). For the time being with available VVI-software, the multiphasic nature of the longitudinal wall motion does not seem to be evaluable. Instead of the approach by Cinthio *et al*. to assess detailed wall motion pattern in a localized region of interest, we observed rather reproducible measurement by averaging wall motion values from a 1-cm vessel segment and thus suggest measuring of peak systolic and diastolic values to provide the most stable vessel wall motions values using current the VVI software.

As a result of the very limited study population and a number of confounders, the difference between the healthy controls and patients with CAD should be interpreted with great caution. Potential clinical relevance of this technique remains to be addressed in a much larger and more homogenous clinically relevant population in the future.

## Conclusions

This study demonstrates CCA longitudinal wall motion to be measurable with VVI-technique with acceptable accuracy. Patients with established CAD appear to exhibit impaired longitudinal wall motion compared to healthy volunteers. Future studies should be directed to further address the longitudinal wall motion, regarding its clinical correlates and potential role as a novel vascular biomarker for cardiovascular diseases.
